# Comment on “Association of low muscle mass index and sarcopenic obesity with knee osteoarthritis: a systematic review and meta-analysis”

**DOI:** 10.1080/15502783.2025.2470230

**Published:** 2025-02-28

**Authors:** Jinxiang Peng, Haozhu Chen

**Affiliations:** Hubei Enshi College, Medical Department, Enshi, China; Hubei University of Chinese Medicine, College of Acupuncture and Bone Injury, Wuhan, China

The article written by Qiming Wu and others on the association between Association of Low Muscle Mass Index and Sarcopenic Obesity with Knee Osteoarthritis: A Systematic Review and Meta-Analysis [1], published in the 2024 issue of the journal “Journal of the International Society of Sports Nutrition.” The systematic review and meta-analysis aimed to investigate the relationship between muscle mass loss and knee osteoarthritis, a common age-related disease with significant public health implications. The study included 12 articles, providing evidence that low muscle mass index and sarcopenic obesity are associated with an increased risk of developing knee osteoarthritis. The significance and value of this article lie in its comprehensive examination of the association between low muscle mass index and sarcopenic obesity with knee osteoarthritis, a disease that poses a substantial burden on public health. By providing high-quality evidence through a meta-analysis, this study aids in identifying modifiable risk factors and potential therapeutic targets, which is crucial for developing prevention and treatment strategies. While recognizing the value of this study, we would like to express the following opinions.

First, a major limitation of this study is the inability to infer causality due to the cross-sectional nature of the included studies. Mendelian randomization (MR) analysis is recommended for future studies to strengthen causal inferences and mitigate the effects of confounding and reverse causality [2,3]. A two-sample Mendelian randomization design, employing genetic instrumental variables strongly associated with low muscle mass index (such as ALM/height^2^ and knee osteoarthritis (KOA) is suggested. In particular, gene loci related to muscle synthesis or degradation should be utilized. Furthermore, future studies should obtain data from public databases, such as the UK Biobank, to verify causal associations [4].

Second, while the article conducted subgroup analyses for cross-sectional and cohort studies, it did not specify the methods for interaction tests between these subgroups. Future studies should use statistical techniques such as the Cochran’s Q test for heterogeneity and test for interactions using stratified analyses or meta-regression models to evaluate potential effect modifications across different study designs [5].

Additionally, the original study does not address heterogeneity or publication bias. Future studies should consider assessing heterogeneity across studies using I^2^ statistics and exploring publication bias through funnel plots or Egger’s test [4]. These steps will improve the reliability and generalizability of the findings.

Our suggestions aim to enhance the already robust research findings, offering additional avenues to refine and strengthen the evidence for future studies.

Finally, the clinical significance of this research lies in its potential to guide future therapeutic strategies for knee osteoarthritis. By identifying the relationship between low muscle mass, sarcopenic obesity, and KOA, the findings can inform interventions aimed at improving muscle health and preventing or delaying the onset of KOA. Early detection and targeted therapies that focus on maintaining muscle mass and strength may reduce the burden of this debilitating disease, ultimately improving patients’ quality of life.

## Originality of manuscript

We affirm that the manuscript is an original work and has not been published previously nor is it under consideration for publication elsewhere.
